# Reorganization of functional brain network architecture in chronic osteoarthritis pain

**DOI:** 10.1002/hbm.25287

**Published:** 2020-11-19

**Authors:** Joana Barroso, Kenta Wakaizumi, Ana Mafalda Reis, Marwan Baliki, Thomas J. Schnitzer, Vasco Galhardo, Apkar Vania Apkarian

**Affiliations:** ^1^ Departamento de Biomedicina, Faculdade de Medicina Universidade do Porto Porto Portugal; ^2^ Instituto de Investigação e Inovação em Saúde ‐ i3S Universidade do Porto Porto Portugal; ^3^ Department of Physical Medicine and Rehabilitation Northwestern University, Feinberg School of Medicine Chicago Illinois USA; ^4^ Department of Physiology Northwestern University, Feinberg School of Medicine Chicago Illinois USA; ^5^ Shirley Ryan Ability Lab Chicago Illinois USA; ^6^ Department of Anesthesiology Keio University School of Medicine Tokyo Japan; ^7^ Unilabs Boavista Porto Portugal; ^8^ Department of Internal Medicine/Rheumatology Northwestern University, Feinberg School of Medicine Chicago Illinois USA; ^9^ Department of Anesthesia Northwestern University, Feinberg School of Medicine Chicago Illinois USA

**Keywords:** brain networks, brain topology, chronic pain, graph properties, osteoarthritis

## Abstract

Osteoarthritis (OA) manifests with chronic pain, motor impairment, and proprioceptive changes. However, the role of the brain in the disease is largely unknown. Here, we studied brain networks using the mathematical properties of graphs in a large sample of knee and hip OA (KOA, *n* = 91; HOA, *n* = 23) patients. We used a robust validation strategy by subdividing the KOA data into discovery and testing groups and tested the generalizability of our findings in HOA. Despite brain global topological properties being conserved in OA, we show there is a network wide pattern of reorganization that can be captured at the subject‐level by a single measure, the hub disruption index. We localized reorganization patterns and uncovered a shift in the hierarchy of network hubs in OA: primary sensory and motor regions and parahippocampal gyrus behave as hubs and insular cortex loses its central placement. At an intermediate level of network structure, frontoparietal and cingulo‐opercular modules showed preferential reorganization. We examined the association between network properties and clinical correlates: global disruption indices and isolated degree properties did not reflect clinical parameters; however, by modeling whole brain nodal degree properties, we identified a distributed set of regions that reliably predicted pain intensity in KOA and generalized to hip OA. Together, our findings reveal that while conserving global topological properties, brain network architecture reorganizes in OA, at both global and local scale. Network connectivity related to OA pain intensity is dissociated from the major hub disruptions, challenging the extent of dependence of OA pain on nociceptive signaling.

## Graphical abstract

Osteoarthritis (OA) manifests with chronic pain, motor impairment and proprioceptive changes. However, the role of the brain in the disease is largely unknown. Here, we studied brain networks using the mathematical properties of graphs in knee and hip OA. We show that while conserving global topological properties, brain network architecture reorganizes in OA, at both global and local scale. Network connectivity related to OA pain intensity is dissociated from the major hub disruptions found:insula/M1/S1/parahippocampus; challenging the extent of dependence of OA pain on nociceptive signaling.

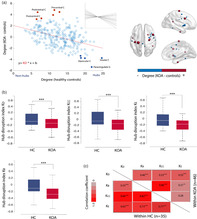

## 1 INTRODUCTION

Osteoarthritis (OA) is one of the most prevalent sources of chronic musculoskeletal pain (Neogi, 2013), and a leading cause of disability worldwide (Centers for Disease Control and Prevention, 2009; Murray et al., 2012). Pain is the hallmark symptom of the disease, contributing to reduced mobility and psychological stress, and representing the main motivation to seek medical care (Hawker, 2009). When compared to other chronic painful conditions, OA pain is unique in numerous ways: there is an extraordinary interpatient variability in clinical symptoms and structural manifestations, and a heterogeneous response to treatment interventions (Deveza, Nelson, & Loeser, 2019). It is not clear to what extent pain is a reflection of afferent nociceptive signaling, represents central adaptive or maladaptive processes, and/or is contingent on psychological and emotional dimensions (Neogi, 2013). Evidence derived from rodent models of OA shows nervous system reorganization, mostly highlighting peripheral changes in nociceptors and spinal cord circuitry (Miller et al., 2015; Miller, Block, & Malfait, 2017); however, the contribution of the brain in the development and progression of OA pain remains minimally explored.

Individual brain regions are functionally specialized, and information exchange between brain areas is fundamental for integrated perceptual states. All cognitive‐emotional functions require such integration (van den Heuvel & Sporns, 2013). Thus, functional resting‐state brain network properties reflect both disease and normal abilities of information integration in the brain. By describing brain networks as graphs, essentially comprising sets of nodes (neuronal elements/brain regions) and edges (their interconnections), we can study key organizational features of the brain's network architecture: locally, at the each node level; globally, both by considering whole‐brain mean nodal properties, and by calculating graph disruption indices—global metrics sensitive to the reorganization of nodes within the network (Mansour et al., 2016).

Using this theoretical framework, we (Huang et al., 2019; Mansour et al., 2016) and other groups (De Pauw et al., 2020; Kaplan et al., 2019) have studied brain networks' global topological properties in chronic pain. It has been shown, for diverse clinical conditions, that CP is associated with large‐scale brain functional changes. Specifically for OA pain, we have recently shown that brain gray matter distortions are distributed across cortical and subcortical structures and relate to pain correlates (Barroso et al., 2020). Moreover, differences in brain modular organization in OA were previously reported in the insula and parietal cortices (Mansour et al., 2016), and the anterior insula was proposed as a key element driving changes in brain network temporal dynamics in chronic OA pain patients (Cottam, Iwabuchi, Drabek, Reckziegel, & Auer, 2018). Here, we hypothesize that together with a global disturbance, local network metrics should be altered in several regions/networks for which structural/functional properties have shown abnormalities in OA, and moreover, that the community/subnetwork organization should also reflect the disease process.

In addition, there is good evidence that brain‐network organizational properties are closely related to function, both in health and in disease (Stam, 2014). Graph disruption indices and local topological changes were previously related to pain intensity and other dimensions of clinical pain (De Pauw et al., 2020; Kaplan et al., 2019; Mansour et al., 2016). We therefore theorize that global and local network disruptions in OA will relate with pain and other key clinical correlates of the disease, potentially reflecting adaptive and maladaptive brain anatomical and physiological plasticity.

In order to test our hypotheses, using resting state functional MRI data from a large sample of long‐duration, severe OA pain patients, we modeled large‐scale brain networks as graphs. We investigated differences between knee OA (KOA) patients and healthy controls (HC) at multiple levels of network topographic structure. Moreover, we evaluated the associations between brain topological properties and clinical correlates of the disease. To ensure that outcomes were robust and applicable to KOA cohorts at large, we validated our findings in a KOA hold out sample, and further tested generalizability in a hip OA (HOA) sample.

## 2 MATERIALS AND METHODS

### 2.1 Participants

This study included 95 KOA and 24 HOA patients with indication for total arthroplasty and 36 HC participants. Patients were recruited in the Orthopedic Department of *Centro Hospitalar e Universitário de São João*, Porto, Portugal. HC were healthy subjects from the same geographic area, age range, social and educational background as the OA patients.

The following inclusion criteria for OA were applied: (a) age between 45 and 75 years; (b) diagnosis of OA according to the clinical classification criteria of the American College of Rheumatology (4); (c) surgical indication for arthroplasty. Exclusion criteria included: (a) secondary OA due to congenital and development diseases or inflammatory and auto‐immune articular diseases; (b) bilateral OA with indication for contralateral arthroplasty in the following year or bilateral knee pain with less than or equal to four points difference on the numeric pain rating scale (NRS) between knees; (c) other chronic pain conditions (e.g., low back pain; fibromyalgia; chronic pelvic pain; chronic headache/migraine); (d) chronic neurological or psychiatric disease (e.g., multiple sclerosis and other demyelinating diseases; peripheral neuropathy; bipolar and related disorders; neurodevelopment disorders); and (e) previous history of stroke or traumatic brain injury. Controls were included if matching patients demographic characteristics regarding age, gender, and educational level. Exclusion criteria for this group were the same as for patients, in addition to the diagnosis of OA in any joint, or undiagnosed joint pain.

All OA patients were evaluated 2–6 weeks prior to surgery with a clinical examination, questionnaires, and a brain MRI. HC underwent a clinical examination and completed a brain MRI. All subjects provided informed consent prior to participating in the study, and all methods were carried out in accordance with the local Ethics Committee (*Comissão de Ética para a Saúde*, *Centro Hospitalar e Universitário de São João*) and the Helsinki declaration.

After brain imaging quality control, a total of 91 KOA, 23 HOA, and 35 HC were considered for formal analysis. OA patients were subdivided in three groups: KOA discovery (*n* = 46); KOA validation (*n* = 45), here using the Kennard–Stone algorithm (5), which allowed us to select samples with a uniform distribution over a multivariate predictor space (age, sex, pain levels, and behavioral variables); and HOA. The first set, the KOA discovery group, was used in the primary analysis of the paper; the KOA validation and the HOA groups were used to assess the external validity of the main findings.

### 2.2 Clinical parameters

Clinical and demographic parameters were obtained at the initial interview, after consenting the patient, and by clinical chart assessment. All patients completed a battery of questionnaires assessing multiple domains: NRS for pain; Knee and Hip Injury and Osteoarthritis Score (KOOS) (Gonçalves, Cabri, Pinheiro, & Ferreira, 2009; Roos & Toksvig‐Larsen, 2003); Hospital Anxiety and Depression Scale (HADS) (Pais‐Ribeiro et al., 2007; Zigmond & Snaith, 1983); Pain Catastrophizing Scale (PCS) (Azevedo, 2007; Sullivan, Bishop, & Pivik, 1995); *Doleur Neuropathique en 4 questions*—Neuropathic pain scale (DN4) (Azevedo, 2007; Bouhassira et al., 2005). All questionnaires were used in their Portuguese validated versions. We also assessed physical performance by applying two distinct tasks: timed up and go test (TUG) (Podsiadlo & Richardson, 1991) and 6‐min walking test (6MWT) (Balke, 1963; Rejeski et al., 1995). Finally, joint X‐rays were also assessed by two trained radiologists and classified using the Kellgren–Lawrence scale (Kellgren & Lawrence, 1957).

Regarding these parameters, descriptive statistics were used to describe the study sample, with continuous variables presented as mean and *SD*s, and categorical data as numbers and percentages. Comparisons between HC, KOA, and HOA groups were done with analysis of variable and independent sample *t* tests or chi‐square (*X* ^2^) tests, for continuous parametrical variables and categorical data, respectively.

### 2.3 Magnetic resonance imaging data acquisition

For all participants, MPRAGE type T1‐anatomical brain images were acquired with a 3.0 T Siemen Magnetom Spectra scanner (Siemens Medical, Erlagen, Germany). Acquisition parameters: isometric voxel size = 1 × 1 × 1 mm, TR = 2,500 ms, TE = 3.31 ms, flip angle = 9°, in‐plane matrix resolution = 256 × 256, number of slices = 160; field of view = 256 mm.

Resting‐state fMRI (rs‐fMRI) images were acquired at the same session and scanner, with the following parameters: Multi‐slice T2*‐weighted echo‐planar images with repetition time TR = 2,500 ms; echo time TE = 30 ms; flip angle = 90 °; voxel size = 3.4 × 3.4 × 3.0; in‐plane resolution = 64 × 64, number of volumes 300; number of slices = 40 (slices covered the whole brain from the cerebellum to the vertex), with interleaved ordering. The time of acquisition lasted 12 min and 39 s, and patients were instructed to keep their eyes open and to remain still during the acquisition.

### 2.4 rs‐fMRI data preprocessing

Preprocessing of each subject's time series of rs‐fMRI volumes was performed using the FMRIB Expert Analysis Tool (www.fmrib.ox.ac.uk/fsl) (Smith et al., 2004) and in‐house software and encompassed the following steps: discarding the first four (10 s) volumes to eliminate saturation effects and achieve steady‐state magnetization; skull extraction using BET; slice time correction; motion correction; spatial smoothing with a full width at half maximum Gaussian kernel of 5 mm; high pass temporal filtering (0.1 Hz) for correcting low frequency signal drift. Afterward, several sources of noise were removed from the data: we regressed the six parameters obtained by rigid body correction of head motion, global signal averaged over all voxels of the brain, white matter signal averaged over all voxels of eroded white matter region, and ventricular signal averaged over all voxels of eroded ventricle regions. Next, in order to further remove motion artifacts, we performed a motion‐volume censoring procedure: we calculated a composite motion score based on normative thresholds (volumes with frame‐wise displacement larger than 0.5 mm, derivative variance root mean square after Z‐normalization larger than 2.3 and deviation of volume intensity within the predefined gray matter mask larger then 2.3); then, we scrubbed the detected volume (volume = i) and adjacent four volumes (i‐2,i‐1, i, i + 1, i + 2) (Power et al., 2014; Power, Barnes, Snyder, Schlaggar, & Petersen, 2012). Finally, and because we are interested in low‐frequency fluctuations of rs‐fMRI signal, we applied a Butterworth filter (0.008–0.1 Hz) to the scrubbed time‐series. Finally, all preprocessed rs‐fMRI data were normalized to standard MNI space, using nonlinear registration (FNIRT) (ref: https://www.fmrib.ox.ac.uk/datasets/techrep/tr07ja1/tr07ja1.pdf). The registered brains were visually inspected to ensure optimal registration.

### 2.5 Quality control of rs‐fMRI data

To ensure optimal quality of the data after preprocessing a two‐step procedure was performed. First, the number of censored motion values was evaluated as this reflects the extent of a subject's motion during scanning; a subject reached the criterion for exclusion if he/she had less than 200 volumes after censoring (8 min of rs‐fMRI scanning). Mean and *SD* of volume censoring was 30 ± 7.3, and maximal volume censoring was 51. Thus, no subjects were excluded given this criterion.

Second, a functional connectivity outlier detection was implemented: for each subject the resting‐state functional connectivity was calculated across 256 regions of interest (ROIs) (Power et al., 2011), then the correlation coefficients of all subjects in each group (KOA, total sample; HC) was calculated separately; finally the average of each column was calculated, representing the mean correlation coefficient across subjects of the correlation coefficient across the 256 ROIs. Subjects with low average within‐group correlation (<2 *SD*s from the average in each group) were identified as outliers and excluded from the analysis. A total of four KOA patients, one HOA patient, and one HC subject were excluded given this criterion.

### 2.6 Brain graph calculation and construction

**FIGURE 1:**
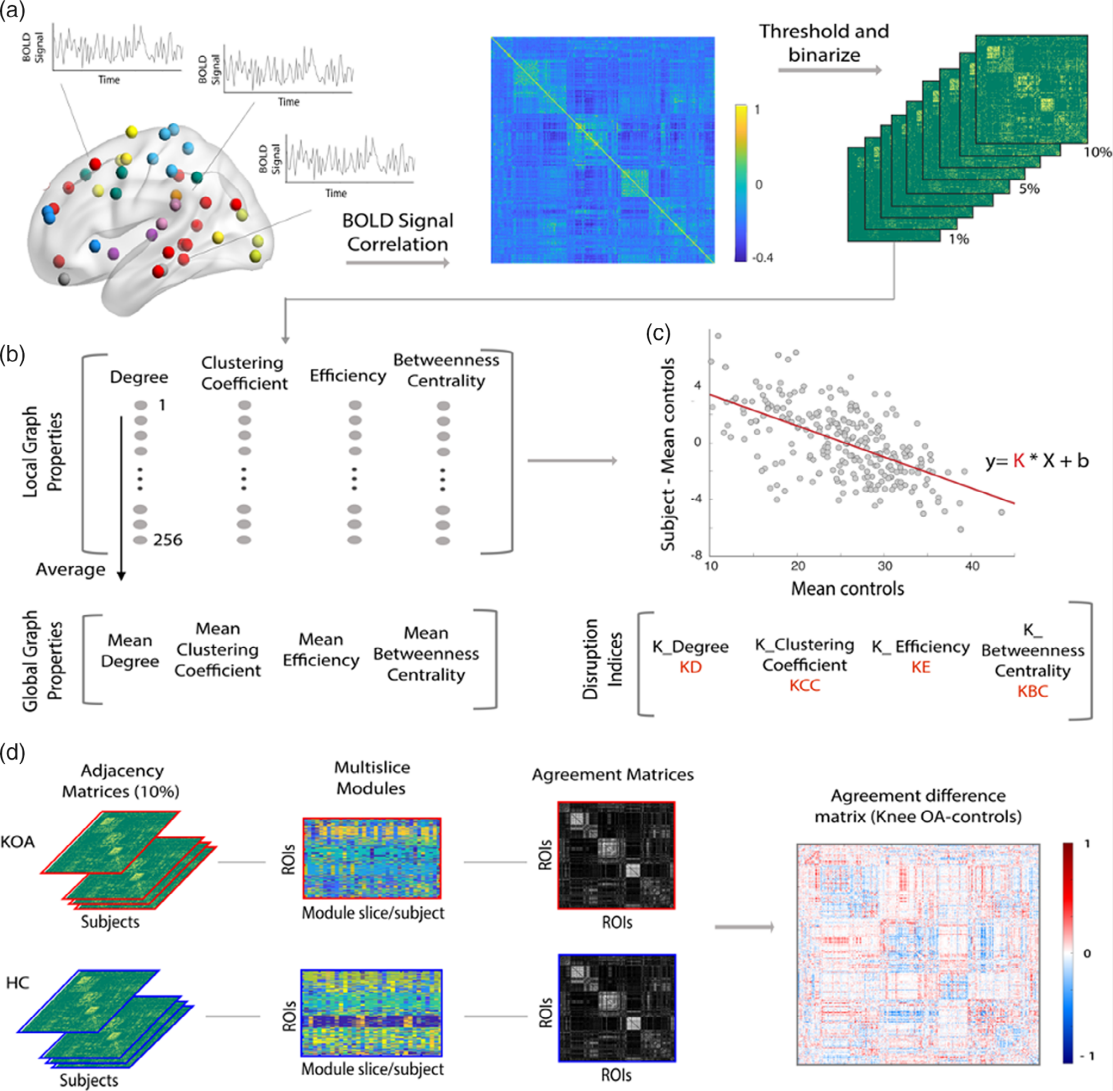
Methodological overview of the computation pipeline for global and nodal graph properties, hub disruption indices and modular reorganization analysis. (a) For each subject included in the study, brain was parcellated in 256 regions of interest (ROIs) from a 264 parcellation Scheme (eight ROIs corresponding to the cerebellum were excluded). For each ROI, blood oxygenation level‐dependent signal (BOLD) was extracted as an average over voxels within 10‐mm diameter spheres with center at defined peak coordinate. Next, a 256 × 256 Pearson's full correlation matrix was computed between all pairs of ROIs time‐series; nine adjacency matrices were then calculated at different link densities (2–10%). (b) Graph properties (degree, clustering coefficient, efficiency, and betweenness centrality) were estimated using the Brain Connectivity Toolbox: First, we calculated nodal (local level) properties; latter, by averaging each property across the 256 ROIs we computed the corresponding global measurement. (c) Hub disruption indices were calculated for each subject as the gradient of a straight line fitted to a scatterplot of the nodal property of interest, for example, degree, minus the same nodal property on average in HC ([osteoarthritis [OA] patient—HC group], y‐axis), versus the mean nodal property in the HC group (x‐axis). (d) Modular reorganization was studied by calculating multislice modularity and agreement matrices separately for knee OA (KOA) and controls (agreement: 0 to 1). A difference agreement matrix was then considered, by subtracting controls agreement matrix to KOA (diff. agreement: −1 to 1). A positive entrance value (red) indicates higher likelihood for the two corresponding ROIs to be in the same module in KOA, but not in the control group. The opposite for negative entrance values (blue). Near zero values reveals pairs of ROIs that behave similarly in both groups. A permutational‐based random model was created by shuffling the two groups over 1,000 times, for further statistical testing

Brain networks can be mathematically described as graphs, comprising sets of nodes (N, ROIs) and edges (M, or connections, here equivalent to interregional pathways). The connectivity structure of a graph is represented by its adjacency matrix; here, an asymmetric binary matrix representing unweighted edges (van den Heuvel & Sporns, 2013). To construct the brain connectivity network for each subject, we followed the approach suggested by Mansour et al. (2016). As shown in Figure 1a, the brain was divided in 256 spherical ROIs, as described by Power et al. (2011), (original parcellation scheme with 264 regions, cerebellum was excluded from the analysis), with 5‐mm radius, located at coordinates showing reliable activity across a set of tasks and displaying a plausible functional structure, spanning the cerebral cortex and subcortical structures. Blood oxygenation level‐dependent (BOLD) signal of each ROI was extracted and linear Pearson's correlations were performed on time courses averaged within each ROI, generating a 256 × 256 correlation matrix for each subject. To avoid arbitrary thresholding, network analysis was conducted on fully connected graphs, with both positive and negative values. These matrices were then thresholded to generate binary undirected graphs with connection density (number of edges proportional to the maximum possible number of edges ((*N* × *N* − 1)/2)) in the range 2–10%.

Following, several topological graph properties were computed using the brain connectivity toolbox (Rubinov & Sporns, 2010), as shown in Figure 1b. Under each link density, distinct topological properties were estimated at each node of each individual graph (local properties): degree (D), captures the number of connections that a node has to other nodes of the graph, and high degree nodes can be considered as centers for information integration; efficiency (E), measures how the information is propagated in the network, with high nodal efficiency reflecting higher information propagation ability; clustering coefficient (CC): represents the fraction of edges (out of all possible) that connect the neighbors of a given node, a measure of segregation; betweenness centrality (BC): represents the number of short paths between all nodes of the network that pass through a given node, a measure of influence. For each subject, at each link density, these metrics were estimated for all 256 nodes—local properties; correspondent global properties are calculated as the average across all nodes (Rubinov & Sporns, 2010).

Two other properties were estimated at a global level—modularity (Q, measure of the decomposability of a graph into several sparsely interconnected structures) and small‐worldness (evaluates the network organization compared to a matched random graph). Modularity was calculated using the Louvain community detection algorithm averaged over 100 repetitions (Rubinov & Sporns, 2010). Small‐world‐ness, based on the tradeoff between clustering and global efficiency (Humphries & Gurney, 2008), is calculated as: ((clustering_*J*_/clustering_random_)/(efficiency_random_/efficiency_*J*_)), where a network is deemed a “small‐world” if the ratio >2.

Differences in global graph properties between groups (KOA and HC) were computed using repeated measures ANCOVA (densities 2–10%), controlling for the effects of age and gender.

### 2.7 Graph topological hub disruption indices

We estimated the hub disruption index, *K*, for the different graph properties computed before: disruption index for degree (*K*_D_), efficiency (*K*_E_), clustering coefficient (*K*_CC_), and betweenness centrality (*K*_BC_), following methods described by Achard et al. (2012). This measure allows us to summarize the abnormal profile of nodal connectivity and topological metrics of an individual subject in relation to the normative topology of the HC group. Figure 1c depicts the computation process. For each subject, we first subtract the HC group mean nodal degree from the degree of the corresponding node in a given individual; next, we plot this individual difference against the HC group mean. The hub disruption index, *K*, is then defined as the gradient of a straight line fitted to the scatter plot following the linear regression (*y* = *K* × x + *b*), where *y* = nodal degree of the subject—mean nodal degree of HC; *x* = mean nodal degree of HC; *b* = residual or intercept of the regression. After computing the individual disruption indices, significant differences between groups in *K*_*D*_, *K*_*E*,_ *K*_*CC*_, and *K*_*BC*_ were calculated at 5% link density using an ANCOVA with age and sex as covariates of no‐interest. We additionally performed a repeated measure ANCOVA accounting for all link densities (2–10%), while controlling for age and sex. Relationships between *K* measures and clinical parameters were estimated using linear partial correlations, controlling for the effect of age and sex. Given the multiplicity of measures (4 hub disruption indices; 12 clinical parameters), FDR correction for multiple comparisons was applied at *α* = .05.

In order to investigate the contributions of regional perturbations to *K* measures, we recomputed individual *K* indices after random removal of 80% of nodes. Pearson's correlation with the original *K* measures was then examined.

### 2.8 Characterization of hub disruption—Nodal statistical analysis

Nodal degree properties were further studied in an effort to better characterize the particular patterns underlying these global changes (*K*_*D*_), and better examine their relationship with clinical properties of the disease. We applied two distinct strategies:

1. We identified nodes that showed a difference in mean degree (*y*, nodal degree of OA group—nodal degree of HC) greater than ±2 *SD* from the mean difference value; we used permutation‐based testing to estimate statistical significance of the between group differences for identified ROIs by randomly assigning subjects to two groups, arbitrarily defining one as the reference and estimating the nodal differences between groups. We repeated this process 10,000 times to sample the null distribution of the nodal group mean difference. After identifying topmost disrupted cortical nodes, we studied their association with clinical parameters of the disease. We then linearly modeled each clinical variable of interest with the identified nodes, applying a stepwise forward and backward selection method, with *α*‐to‐enter set at .05 and *α*‐to‐remove at .10. For all regression models, assumptions of linearity, independence of observations, homoscedasticity, and absence of multicollinearity were met, and residuals were approximately normally distributed.
2. In a more exploratory form, and given that *K* permeates the whole‐brain network, implicating that the disruption is not merely driven by functional changes to specific regions or pathways, we studied the relationship of whole‐brain nodal degree and pain intensity in OA using a L1/L2 regularized linear regression (i.e., elastic net) (Zou & Hastie, 2005). This method allows us to remove predictors with low influence on the outcome while regularizing for enhanced generalization. The coefficients of the nonrelevant features are shrunk toward zero, simplifying the model and reducing overfitting. Using the KOA discovery group, we applied a linear regression with a regularization penalty *α* = .5, to predict pain intensity (NRS), based on the nodal degree properties of all 256 ROIs. Ten‐fold cross‐validation was used to choose the hyperparameter, *λ* (shrinkage parameter), that minimized mean squared error (MSE) over a default grid size of 100; the hyperparameter *λ* that generated the smallest MSE was selected for the final model (Friedman, Hastie, & Tibshirani, 2010). Performance of the model was assessed in the discovery and validation cohorts. All degree nodal properties were adjusted for age and sex prior to these analyses, by linearly regression and adding the adjusted fitted values and residuals for each observation.

### 2.9 Modular reorganization analysis

We studied brain networks at an intermediate scale of organization—community structure or modularity—following methods described by Mano et al. (2018)); this approach is based on community detection in multislice networks (Mucha, Richardson, Macon, Porter, & Onnela, 2010) and focuses on detecting differences in brain network modularity between groups (OA, HC) by calculating a measure of consensus modularity pattern – modularity agreement matrix (AM). Analytical steps are depicted in Figure 1d. First, for each group separately (OA discovery, HC), we use a *categorical* multislice modularity algorithm (Jeub, Bazzi, Jutla, & Mucha, 2011), where the same node is coupled among all subjects (slices), with 10% link density matrices. Coupling strength (ω = 0.1) and modularity resolution (γ = 1.5) are free parameters that were a priori defined based on previous research (Mano et al., 2018; Mucha et al., 2010). This allows us to create a single symmetric AM representing each group, where each entry has a value within [0,1], representing the agreement between pairs of nodes. As the number of subjects in each group was different (OA = 46; HC = 35), and the modularity estimation is a probabilistic procedure, we calculated the AMs 1,000 times, selecting randomly 34 patients for each group, and computed the average across repetitions. Next, having one mean AM per group, we computed the agreement difference matrix: <OA AM> − <HC AM>. Here, values range from [−1,1]; large negative values correspond to nodes that are recurrently part of the same modules in controls but not in the OA group, large positive values represent the opposite. Values close to 0 represent nodes with the same behavior, either with high agreement or low agreement in both groups. In order to attain an overall metric of reorganization per region, the absolute sum of positive and negative contributions was computed per node—nodal modular reorganization.

Finally, following Mano et al. (2018), in order to evaluate the statistical significance, we performed a permutational analysis, were we randomly resampled pain and control subjects into two groups and repeated the full analysis—also 1,000 times. Based on the proportion of times the resampled nodal modular reorganization exceeded the correct value we calculated one‐sided *p*‐values and used a threshold of *p* < .01 to consider significant nodal modular reorganization values.

### 2.10 Analysis validation

We aimed to validate the principal findings from the discovery cohort, specifically determining the hub disruption index significance, determining the hub status of disturbed regions and relationship with clinical properties; we also validated the machine learning algorithm applied for pain intensity prediction and modular reorganization in the KOA and HOA validation groups. Connectivity matrices for the validation groups were created and thresholded using identical procedures as described above. Contrasts requiring HCs were assessed with the same 35 HC participants from the discovery data set.

### 2.11 Software/code

Analysis was performed using MATLAB 2019.b (MATLAB and Brain Connectivity Toolbox release 2019a, The MathWorks, Inc., Natick, MA) and tools from the brain connectivity toolbox (Rubinov & Sporns, 2010). Code for modularity reorganization analysis was adapted from Mano et al (http://doi.org/10.5281/zenodo.1183399) (Mano et al., 2018). Brain figures of ROI and functional connectivity networks were visualized on a surface rendering of a human brain atlas with BrainNet Viewer (http://www.nitrc.org/projects/bnv/).

## 3 RESULTS

### 3.1 Demographic and clinical characteristics

No significant differences for gender, BMI, or smoking habit were seen between the KOA and HC groups (Table 1). However, the HC group had a significantly lower mean age (*t* = −9.27; *p* < .001), supporting the importance of controlling for this variable in further analyses. Social‐cultural variables (educational level; habitation; marriage status) did not show significant differences between groups (*p* > .05 for all). Comparing the validation samples (KOA and HOA) with the KOA discovery sample (demographic, pain related, and behavioral data presented in Table 2), both KOA groups were balanced regarding demographic and clinical outcomes; HOA patients presented shorter pain duration, worse radiographic severity and a lower neuropathic pain score; this group had a younger mean age and a significantly higher number of male subjects.

**TABLE 1:** Demographic and clinical characteristics of osteoarthritis patients (discovery group) and controls. To identify demographic difference between groups ANOVA and *t* tests were performed for continuous data. Chi‐square tests were performed for categorical data

	Controls (*n* = 35)	Knee OA (discovery group; *n* = 46)	*p*‐Value
Age (years), mean, *SD*	59.5 ± 7.91	65.3 ± 7.41	.001**
Sex (female), *n*, %	20; 57.1%	30; 65.2%	.49
BMI (kg/m^2^), mean, *SD*	28.2 ± 4.57	30 ± 4.3	.07
Smoking, *n*, %	7; 20%	4; 8.7%	.19
Education, *n*, %			.09
Primary education	20; 57.1%	34; 73.9%	
Secondary education	8; 22.9%	8; 17.4%	
Postsecondary education	7; 20%	4; 8.7%	
Habitation, *n*, %			.78
Alone	6; 17.4%	10; 21.7%	
Cohabitation	29; 82.8%	36; 78.3%	
Marital status, *n*, %			.28
Married	26; 74.3%	30; 65.2%	
Never married	3; 8.6%	3; 6.5%	
Divorced	2; 5.7%	4; 8.7%	
Widowed	4; 11.4%	9; 19.6%	

*Note: p* < .01.

Abbreviations: ANOVA, analysis of variable; BMI, body mass index; OA, osteoarthritis.

**TABLE 2:** Demographic and clinical characteristics of osteoarthritis patients: discovery and testing groups

	Knee OA (discovery group; *n* = 46)	Knee OA (testing group; *n* = 45)	*p*‐Value	Hip OA (testing group; *n* = 23)	*p*‐Value
Age (years), mean, *SD*	65.3 ± 7.41	65.78 ± 5.6	.75	59.5 ± 7.41	.007*
Sex (female), *n*, %	30; 65.2%	38; 84%	.035*	8; 40%	.008*
BMI (kg/m^2^), mean, *SD*	30 ± 4.3	30.87 ± 5.54	.41	28.8 ± 3.36	.08
Education, *n*, %			.15		.76
Primary education	34; 73.9%	38; 84.4%		14; 70%	
Secondary education	8; 17.4%	6; 13.3%		4; 20%	
Postsecondary education	4; 8.7%	1; 2.2%		2; 10%	
Smoking, *n*, %	4; 8.7%	3; 6.7%	.71	2; 8.7%	1.0
Habitation, *n*, %			.59		.52
Alone	10; 21.7%	7; 15.6%		3; 13%	
Cohabitation	36; 78.3%	38; 84.4%		20; 90%	
Marital status, *n*, %			.19		.57
Married	30; 65.2%	35; 77.8%		16; 70%	
Never married	3; 6.5%	2; 4.4%		2; 8.7%	
Divorced	4; 8.7%	3; 6.7%		2; 8.7%	
Widowed	9; 19.6%	5; 11.1%		3; 13%	
NRS, mean, *SD*	6.48 ± 1.42	6.8 ± 1.93	.37	6.2 ± 1.59	.21
DN4, mean, *SD*	2.7 ± 2.26	2.9 ± 2.15	.55	1.82 ± 1.49	.025*
HADS_D, mean, *SD*	7.9 ± 3.93	6.7 ± 4.33	.15	5.8 ± 4.45	.46
HADS_A, mean, *SD*	8.6 ± 4.07	9 ± 5.71	.71	6.5 ± 4.19	.07
HOOS_S, mean, *SD*	60.5 ± 19.31	61.9 ± 21.8	.74	50 ± 16.02	.023*
HOOS_P, mean, *SD*	63.6 ± 16.52	64.2 ± 16.43	.87	55 ± 17.75	.063
HOOS_ADL, mean, *SD*	61.4 ± 15.84	64.4 ± 17.54	.38	57.9 ± 18.3	.15
HOOS_SR, mean, *SD*	91.5 ± 17.63	91.6 ± 15.75	.97	83.15 ± 19	.054
HOOS_QL, mean, *SD*	78.6 ± 16.37	80 ± 17.46	.68	70.6 ± 20.4	.051
PCS, mean, *SD*	19.1 ± 12.33	23.3 ± 15.75	.15	18.1 ± 11.01	.16
Pain duration (years), mean, *SD*	6.8 ± 5.45	8.5 ± 6.44	.61	5.03 ± 4.2	.021*
Radiographic KLS, *n*, %			.33		<.001*
Grade 1	1; 2.2%	—		—	
Grade 2	12; 26.1%	9; 20%		—	
Grade 3	21; 45.7%	22; 48.9%		5; 21.7%	
Grade 4	12; 26.1%	14; 31.1%		18; 72.2%	
STUG, mean, *SD*	13.12 ± 3.93	13.9 ± 4.8	.41	14.26 ± 4.46	.77
6MWT, mean, *SD*	257.8 ± 75.5	272.4 ± 68.93	.33	271.7 ± 99	.9

*p<.05.

Abbreviations: 6MWT, 6‐min walking test; ADL, activities of daily living; ANOVA, analysis of variable; BMI, body mass index; DN4, Douleur Neuropathique en 4 questions; HADS, Hospital anxiety and Depression Scale; HOA, hip OA; HOOS, Hip Injury and Osteoarthritis Outcome Score; KL, Kellgren–Lawrence scale; KOA, knee OA; KOOS, Knee injury and Osteoarthritis Outcome Score; NRS, numeric pain rating; OA, osteoarthritis; PCS, Pain Catastrophizing Scale; QoL, quality of life; SR, sports and recreation; STUG, stand up and go test.

*Note: p*‐Values represent statistical tests between KOA discovery groups and the holdout testing samples; KOA testing sample showed a significantly higher number of females, no other differences were captured; HOA group was significantly different regarding age, gender, DN4 scale, pain duration, and radiographic severity (KLS). ANOVA and *t* tests were performed for continuous data. Chi‐square tests were performed for categorical data.

### 3.2 Global connectivity and network topology properties are not altered in KOA

There were no statistically significant differences between groups (KOA vs. HC) on global measures of network topology as depicted in Figure S1. At every density of functional connections tested (1–10%), functional networks had similar clustering coefficient, global efficiency, betweenness centrality, and modularity, measures reflecting distinct network properties (segregation, integration, centrality, community structure). Both groups' networks exhibited characteristic small‐world organization (high clustering combined with high global efficiency). Hence, despite the clinical differences, global network topological measures were conserved in KOA patients when contrasting to HC.

### 3.3 Disruption of hub organization in OA patients

**FIGURE 2:**
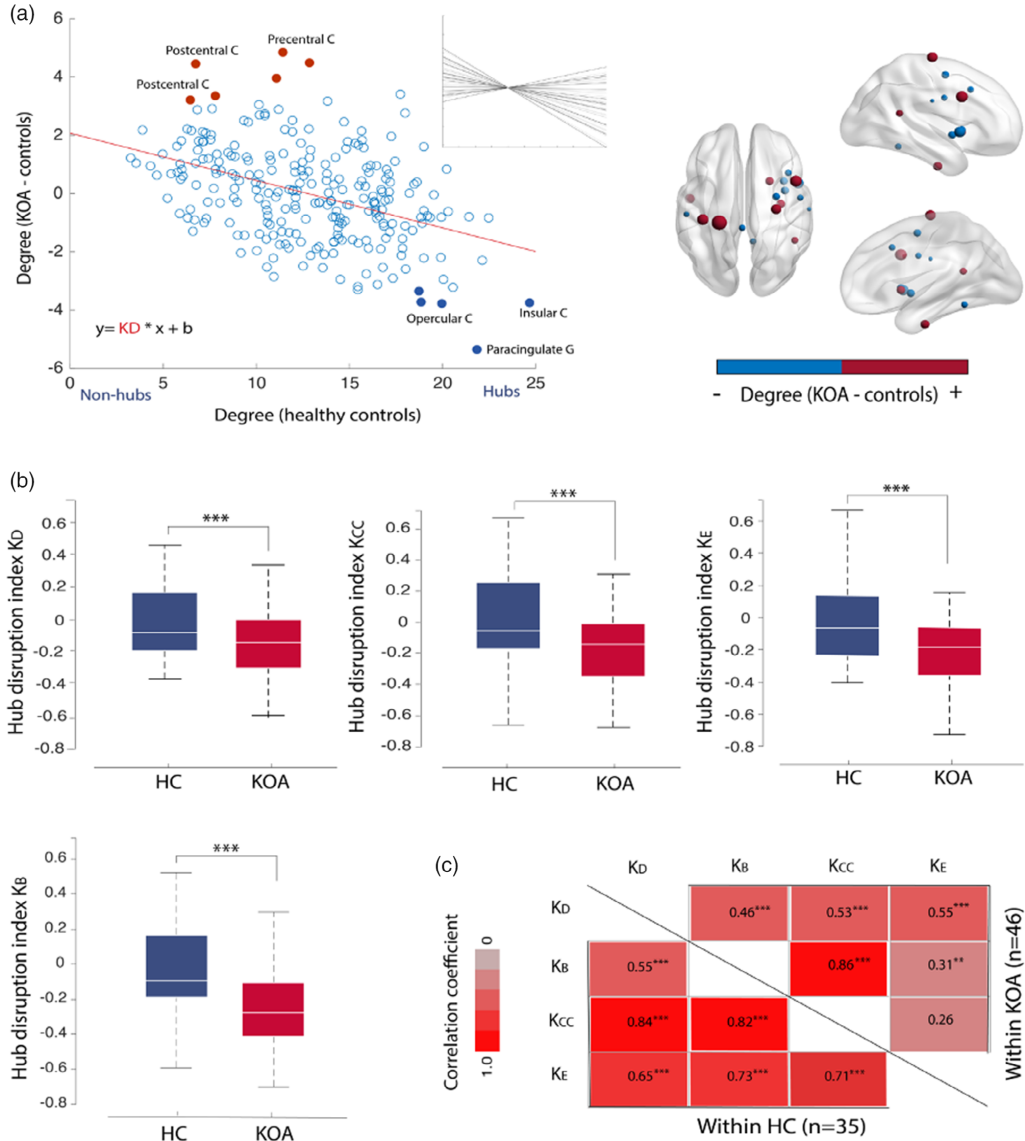
Disruption of functional network hub organization in osteoarthritis (OA) patients. (a) Group hub disruption index calculation for degree (*K*_D_) at 5% link density: for each node (256 regions), the mean degree in the control group (x‐axis) is plotted against the mean nodal difference between groups (KOA—control) (y‐axis). Red dots represent nodes that are non‐hubs in controls but show an abnormal increase in degree in KOA patients: precentral and postcentral gyrus; filled blue dots represent nodes that are typically hubs in healthy controls and show a reduction in degree for KOA: insula; paracingulate gyrus; opercular cortex. The hub disruption index corresponds to the slope of the line fitted to the data (red line), *K*_D_ = −0.18, *p* < .001. Insert shows individual *K*_D_ values. On the right, brain graphical representation of the difference in mean degree between KOA and controls, top 10% most different regions of interest (ROIs) are depicted, red denotes abnormally increased degree and blue abnormally decreased degree in KOA compared to healthy controls. (c) Boxplots of the subject‐wise estimated hub disruption indices for the control group (blue) and KOA (red) at 5% link density. Between‐group differences in *K*_D_, *K*_BC_, *K*_E_, and *K*_CC_ were deemed significant by an ANCOVA (*p* < .001), while controlling for age and gender. Corresponding results for the same measures different graph connection densities are shown in Figure S2. (d) The four hub disruption indices here significantly correlated with each other (Person's r), and the strength of correlation was overall higher in healthy controls than in KOA patients. ***p < .001; **p < .01; HC, healthy control; KOA, knee osteoarthritis

As previous research in diverse clinical conditions has demonstrated that even though global properties of a network may be preserved, nodal properties are reorganize (Achard et al., 2012), we queried if local level disruptions were present in OA patients. We calculated hub disruption indices, a metric that allows to summarize and visualize the pattern of nodal abnormalities. Figure 2a illustrates the results of this metric at group level for degree; rank order for nodal degree is disrupted, with some nodes showing abnormal decrease and others abnormal increase in degree properties. This disruption pattern is summarized by the gradient of a straight line fitted to the data (*K*_*D*_ = −0.18; *p* < .001). Other topological measures exhibited similar results for group‐averaged maps (Figure S2). Next, we assessed hub disruption at an individual level, for all our subjects; Figure 2b shows between‐group differences (HC; OA) of the individually estimated indices; these were statistically significant (*p* < .001) at 5% link density networks, and this result was further validated across other graph connection densities (Figure S3). The four hub disruption indices were significantly correlated with each other, and the strength of correlation was higher overall in HCs than in KOA patients (Figure 2c).

We then assessed the influence of regional perturbations to the magnitude of the disruption indices. At 5% link density networks, individual *K* values were recomputed after random removal of 80% of the nodes (*K*″), and this process repeated 100 times. Mean *K*″ was significantly correlated with K across all topology measures (*p* < .002; *r* values: .58–.81) (Figure S4). These results imply rank order disruption permeates the whole brain network and is not exclusively a product of specific regional perturbations.

### 3.4 Hub disruption indices do not strongly associate with clinical properties of OA

Following previous research, where key clinical properties of a disease were reflected in the hub disruption indices (De Pauw et al., 2020; Itahashi et al., 2014), we studied the relationship between *K* indices and numerous clinical properties of the disease: pain intensity and quality, anxiety and depression, pain catastrophizing, disability, and motor skill tests. We calculated Pearson's partial correlations, controlling for age and sex. As shown in Figure S5, only *K*_CC_ presented a significant association with pain catastrophizing (*r* = .3, *p* = .043) and KOOS symptoms subscale (*r* = .33, *p* = .025); however, these did not survive FDR correction for multiple comparisons (at *p* = .05).

### 3.5 Localized hub topology alterations in OA and its relationship with clinical endpoints

**FIGURE 3:**
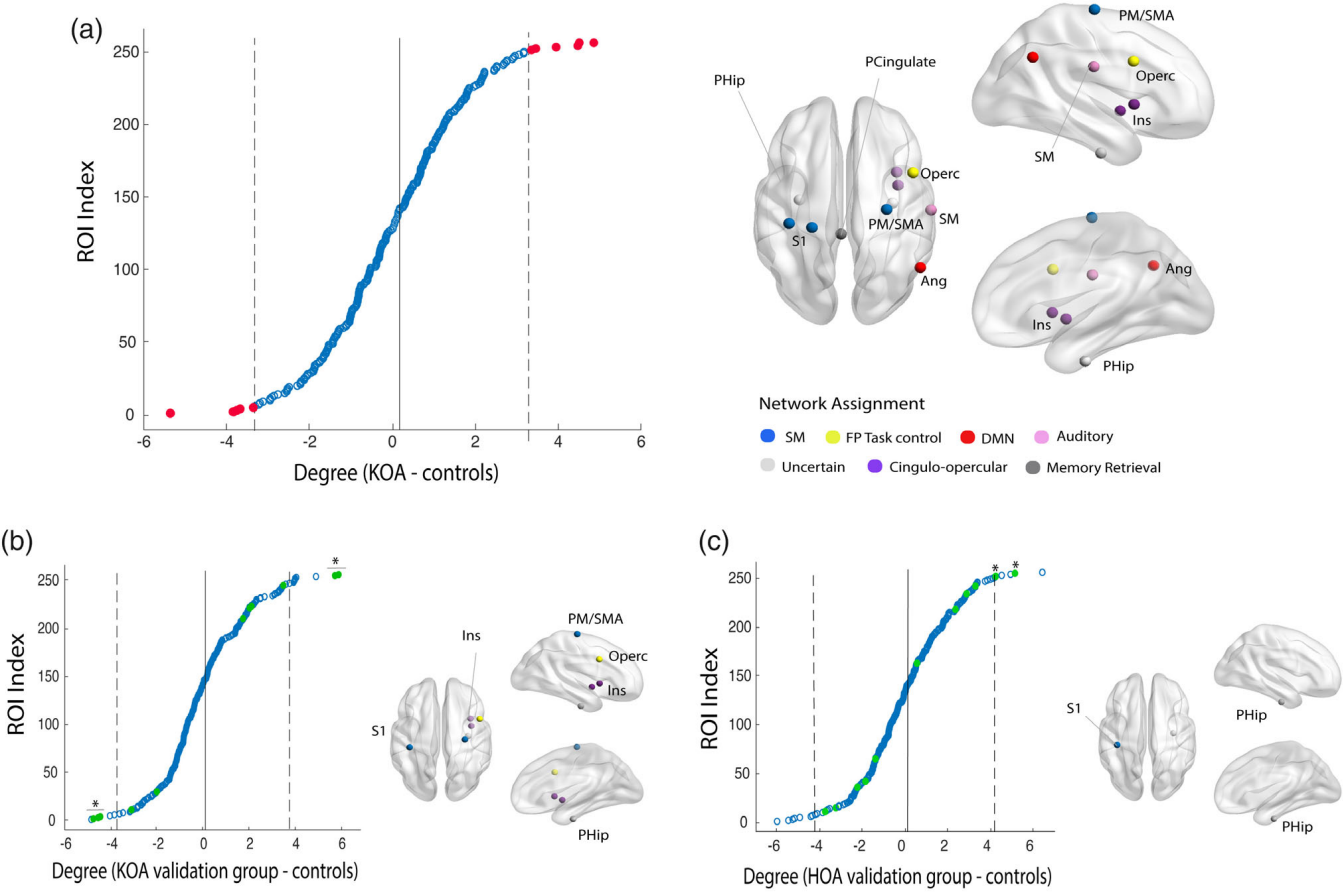
Hub topology is altered in knee OA (KOA): differences in hub status between osteoarthritis (OA) patients and healthy controls. (a) Mean nodal difference between groups (KOA—control), organized by score and thresholded at ±2 *SD* (gray lines) from the mean difference (red dots) and graphic representation of selected nodes. (b) Validation of nodal disruption in KOA hold out sample: 6 out of 11 regions were validated: sensory‐motor regions and parahippocampal gyrus present a significant degree gain and insula/operculum, normal hub nodes, show an abnormal reduction of degree in KOA patients. (c) Hip OA group, validates uniquely the increase in degree for S1 and parahippocampal gyrus. FP, frontoparietal cortex; DMN, default mode network; ROI, region of interest; S1, primary somatosensory cortex; SM, sensory‐motor cortex. *p < .05, permutational test

To better characterize the particular patterns underlying global changes rendered in the *K* metrics, we explored changes at a nodal level by selecting degree, which is the simplest and most commonly used means of identifying hubs (Power, Schlaggar, Lessov‐Schlaggar, & Petersen, 2013) and upon which many other graph properties are based. We started by identifying nodes displaying between group degree differences greater than ±2 *SD* (Figure 3a; Table 3), hereby categorized as top disrupted nodes. Statistical significance of the differences was analyzed with permutational testing. Moreover, significant differences were validated in a subset if nodes in the knee and hip holdout samples (Figure 3b; Table 3): two nodes localized in the insular cortex and one node located in the postcentral gyrus showed consistent loss of hubness in KOA patients; abnormally increased hubness was reliably observed in the precentral gyrus and postcentral gyrus (both nodes assigned to the sensorimotor [SM] network) in KOA/ HOA and temporal fusiform/parahippocampal gyrus in KOA patients compared to HC. Hence, there was a gain in degree in primary motor and sensory nodes that was accompanied by a loss of degree in characteristic associative regions in KOA.

**TABLE 3:** Differences in hub status between OA patients and healthy controls

Difference in mean degree	Coordinate in MNI space	Harvard‐Oxford cortical and subcortical structural atlas	Network assignment^a^	*p*‐Value^b^ 10,000 permutations
(Patient < control)				
5.36	(−2, 35, 31)	Paracingulate cortex, L (68%)	Memory retrieval	< .001
3.77	(52, −59, 36)	Lateral occipital cortex, R (51%)	DMN	.027
3.75	(36, 10, 1)	Insular cortex, R (47%)	CO task control	.04^c^
3.72	(59, −17, 29)	Postcentral gyrus, R (45%)	Auditory	.071
3.35	(37, 1, −4)	Insular cortex, R (53%)	CO task control	.025^c^
(Patient > control)				
4.84	(29, −17, 71)	Precentral gyrus, R (35%)	SM	.006^c^
4.49	(−38, −27, 69)	Postcentral gyrus, L (51%)	SM	.009^c^ ^,^ ^d^
4.45	(−31, −10, −36)	Temporal fusiform gyrus, L (32%); parahippocampal gyrus, L (29%)	Uncertain	.01
3.95	(47, 10, 33)	Precentral gyrus, R (37%)	FP task control	.022
3.41	(−23, −30, 72)	Postcentral gyrus, L (36%)	Uncertain	.044
3.35	(33, −12, −34)	Temporal fusiform gyrus, R (40%), parahippocampal gyrus R (23%)	Uncertain	.026^c^ ^,^ ^d^

Abbreviations: CO, cingulo‐opercular; DMN, default mode network; FP, frontoparietal; MNI, Montreal Neurological Institute; OA, osteoarthritis; SM, sensory‐motor.

*Note:* Regions identified in Figure 3. Peak coordinates (x,y,z) are displayed according to MNI atlas, labels accordingly to the Oxford‐Harvard Structural Cortical Atlas (http://fsl.fmrib.ox.ac.uk/fsl/fslwiki/Atlases).

^a^ Network assignment in accordance with Figure 3.

^b^ Statistical significance of difference was tested under 10,000 permutational tests.

^c^ Nodal differences were further validated in the KOA holdout sample at p<0.05.

^d^ HOA group at *p* < .05 (Figure 3).

Given the consistent disruption of nodal topology, we examined if hubness changes in these regions reflected clinical properties of KOA. Hierarchical multiple regression models were built for each clinical variable of interest (i.e., pain duration; pain intensity; HADS anxiety; HADS depression; PCS; KLS; 6MWT; TUG; DN4; and KOOS subscales). Two models yielded statistical significance: worse ratings in KOOS/Sports and recreation subscale was related to loss of hubness in the postcentral gyrus (*F*(46,44) = 6.98, Adj *R* ^2^ = .11, *p* = .001; *sβ* = −.65); worse scores in TUG were related also to loss of hubness in these regions (*F*(46,45) = 6.42, Adj *R* ^2^ = .1, *p* = .014; *sβ* = −.2). These associations were not validated in the KOA hold out sample (linear regression for selected nodes: KOOS/SR: *F*(45,43) = 0.2, *p* = .65; TUG: *F*(45,43) = 0.4, *p* = .56), and were not tested in the HOA sample (as the identified regions were not validated in the KOA sample).

### 3.6 Whole brain distributed changes in degree predict pain intensity in KOA

**FIGURE 4:**
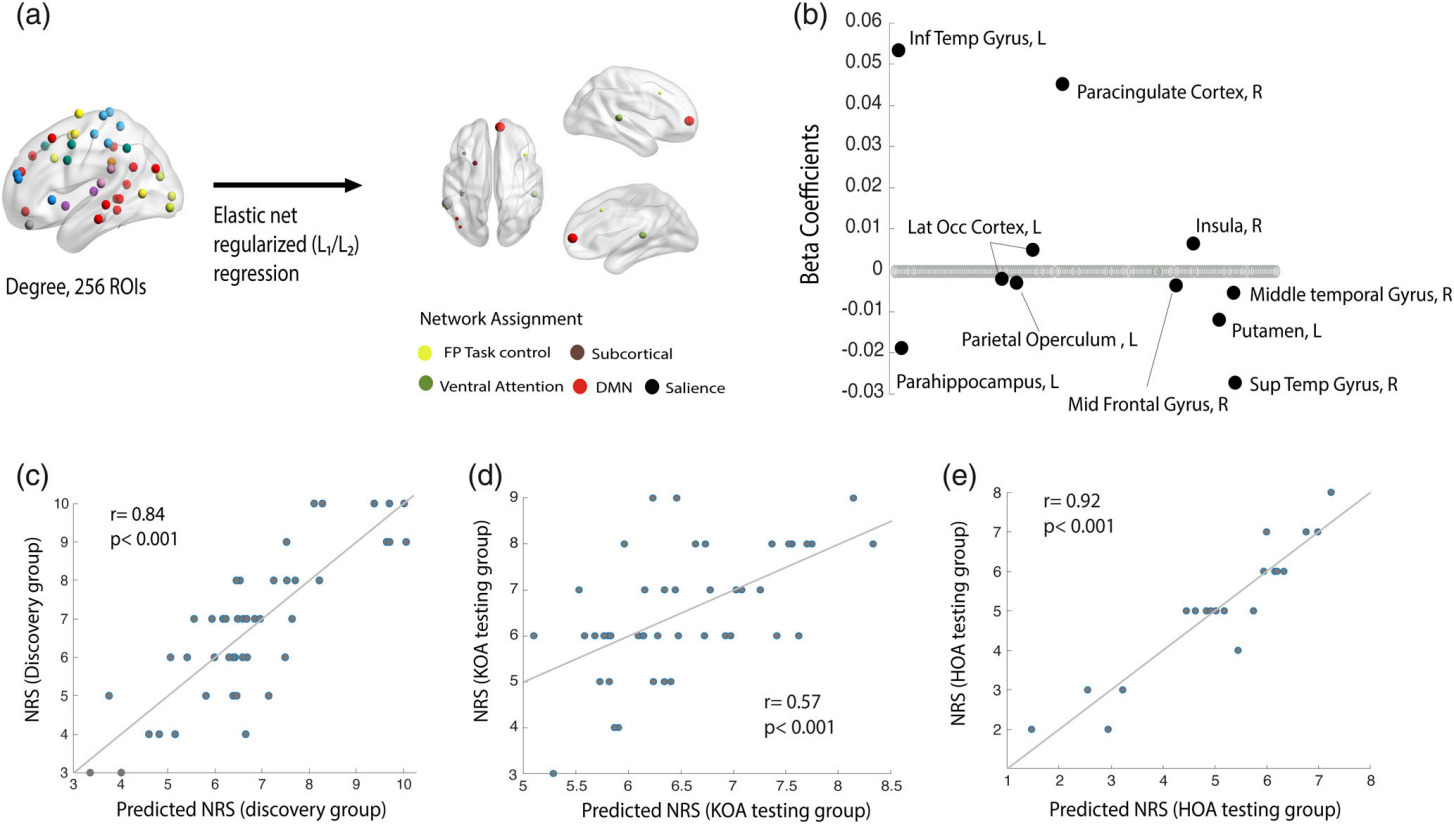
Multinodal distributed degree properties predict pain intensity numeric pain rating (NRS) in osteoarthritis (OA) patients. (a) Graphical representation of the linear regression model using Elastic net regularization and variable selection with penalty weight (*α*) of .5 and regularization parameter (*λ*) choice via a 10‐fold cross validation. Brain nodes depicted correspond to regions predicting pain intensity; node size reflects the weight (B‐coefficients) in the regression model. This is also illustrated in (b) the majority of nodes has regression coefficients set to zero, indicating that the corresponding variables are not contributing to the model. Nonzero regression coefficients identify the predictive features and indicate the weight and direction of degree change in relation to the response variable: that is, higher levels of pain (NRS) relate with lower degree in parahipoccampal gyrus, putamen, and superior temporal gyrus and higher degree in paracingulate cortex and inferior temporal gyrus. (c) Features selected in the elastic net regression predicted the magnitude of response and (d) validate in the hold out knee OA (KOA) and (e) hip OA samples: high correlation value between predicted and actual NRS scores in the KOA discovery group (Pearson's *r* = .84, *p* < .001) KOA holdout testing group (*r* = .57, *p* < .001) and HOA holdout testing group (*r* = .92, *p* < .001)

Given that the top disrupted nodes partially explained only two clinical properties of the disease, that the association was weak and did not generalize and, moreover, given that *K*_D_ permeates the whole brain network, we hypothesized that smaller, distributed changes in nodal reorganization could explain clinical properties of OA. We limited the analysis only to clinical pain intensity (NRS scale); we applied a linear regression using all 256 nodal degree properties as predictors; to remove predictors with low impact to the outcome while improving generalization, we used an elastic net regularized regression. As shown in Figure 4a,b, the combination of 12 brain nodal degree properties, both with increased (i.e., inferior temporal gyrus; paracingulate cortex; insula; lateral occipital cortex) and decreased degree (i.e., putamen; operculum; middle frontal gyrus; parahippocampus) were strong predictors of pain intensity in KOA (Figure 4c predicted/real NRS Pearson's *R* = .83); this result was validated in the KOA hold out sample (predicted/real NRS Pearson's *R* = .57), as shown in Figure 4d. and replicated in HOA (predicted/real NRS Pearson's *R* = .93), Figure 4e. Thus, we show that small, distributed changes in network information‐sharing map pain intensity in KOA, and this result is valid and generalizes to HOA.

### 3.7 Modular reorganization affects mainly regions classified in the frontoparietal and cingulo‐opercular networks

To study brain network reorganization at an intermediate level, we evaluated modularity. Modularity assesses how well a network can be divided into a set of sparsely interconnected subnetworks or modules. We hypothesized that despite the fact that global modularity (Q) is not different between groups (Figure S1); the modular structure could be perturbed in OA, with regard to the identity of nodes making up the different modules.

**FIGURE 5:**
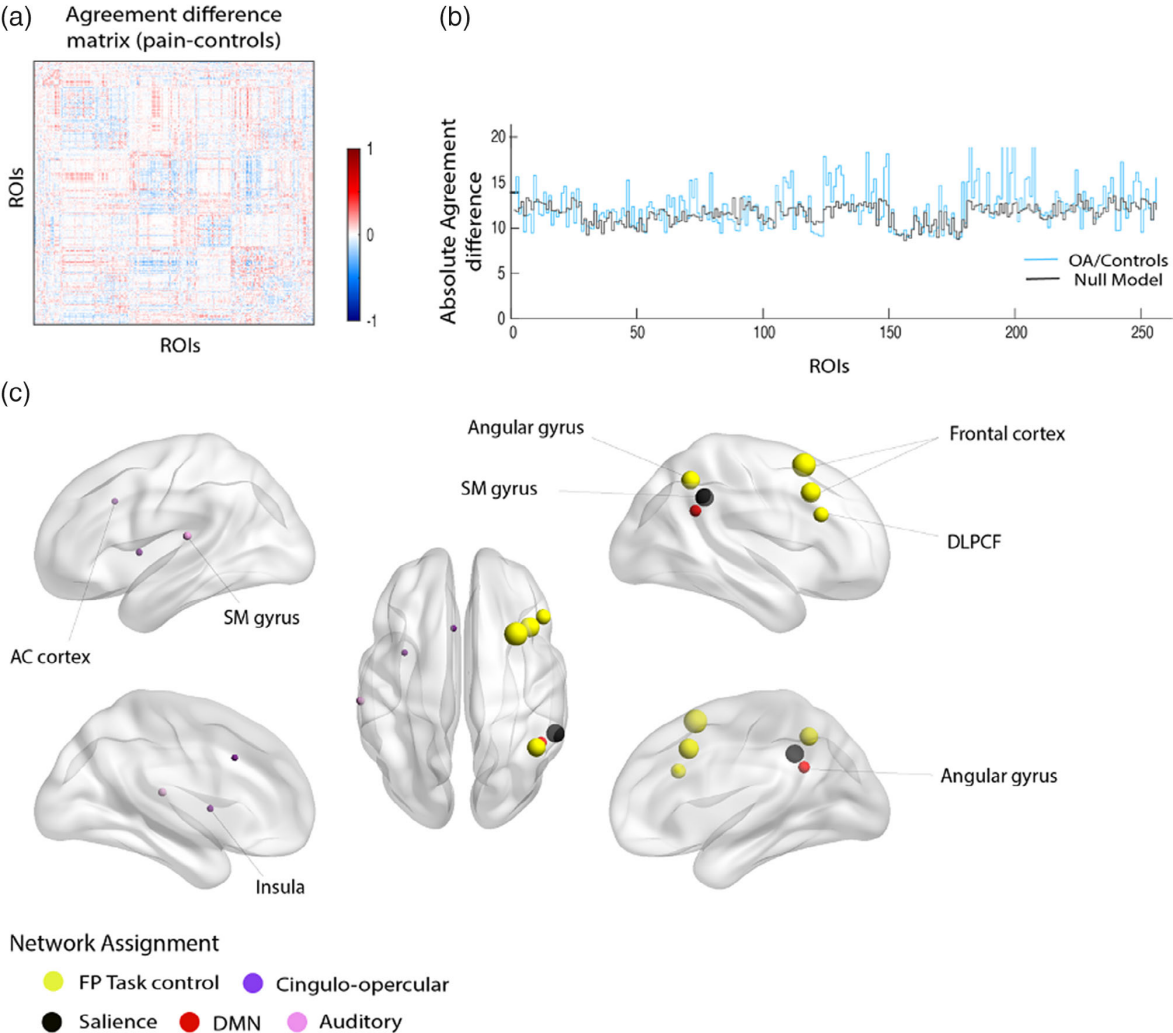
Modular reorganization in osteoarthritis (OA) involves predominantly nodes assigned the frontoparietal and cingulo‐opercular task control networks. (a) Agreement difference matrix obtained from the difference between the modular agreement matrix from OA and control groups. Positive (red) values reflect pairs of nodes that are estimated to appear more commonly in the same module in OA patients, and negative (blue) values represent pairs of nodes that are estimated to appear less commonly in the same module in OA. White value represents regions of interest (ROIs) that has the same behavior in both groups. (b) Blue line shows overall modular reorganization for each node as the sum of both positive and negative values for each node (sum of absolute value per row in (a)), meaning the largest value, the greater reorganization. To statistically evaluate these values, we performed a permutation test of sum reorganization estimation (null model, gray color), yielding one‐side *p*‐values across all ROIs. (c) Representation of statistically significant nodes showing modular reorganization at a threshold of *p* < .01; size reflects magnitude of the absolute agreement difference

Closely following methods recently described (Mano et al., 2018), we estimated a modularity consensus or AM for each group (OA, HC), and computed the difference between group matrices, generating the agreement difference matrix (Figure 5a). Here, each entry takes a value from 1 (red), to −1 (blue). Large positive values identify nodes that appear more commonly in the same network in pain patients than in controls and large negative values relate to pairs of nodes that are less likely to be part of the same network in those patients. Entries close to zero identify regions that have the same behavior in both groups. By creating an index per node, the absolute sum of values per row, defines its overall modular reorganization. Figure 5b represents the following steps, where statistical significance for modular reorganization indices was tested against a null model. Figure 5c illustrates the regions after thresholding at *p*‐value of .01. Nodes showing larger modular reorganization were located mainly in the middle frontal gyrus, part of the frontal–parietal task control network. Supramarginal gyrus, angular gyrus and regions located at the cingulate cortex, insula and operculum were also identified. When validating our findings in the KOA and HOA holdout groups, regions located in the frontoparietal and cingulo‐opercular network were validated (Table 4).

**TABLE 4:** Brain regions showing modular reorganization after permutational‐based analysis against a random model, at a cut‐off threshold of *p* < .01

Absolute agreement difference (+/−)	Coordinate in MNI space	Harvard‐Oxford cortical and subcortical structural atlas	Network assignment^a^	*p*‐Value^b^ 1,000 permutations
23.28 (10.27, −13.01)	(44, −53, 47)	Angular gyrus, R (40%)	FP task control	<.001
26.25 (12.84, −13.39)	(32, 14, 56)	Middle frontal gyrus, R (42%)	FP task control	<.001^c^ ^,^ ^d^
23.35 (10.14, −13.21)	(55, −44, 37)	Supramarginal gyrus, R (43%)	Salience	<.001
21.45 (10.59, −10.87)	(48, 25, 27)	Middle frontal gyrus, R (39%)	FP task control	.002
24.31 (10.56, −13.76)	(39, 18, 39)	Middle frontal gyrus, R (50%)	FP task control	.004^b^
17.15 (8.14, −9.01)	(−60, −25, 14)	Parietal operculum cortex, L (35%)	Auditory	.004
16.46 (6.97, −9.48)	(−5, −18, 34)	Cingulate gyrus, L (29%)	CO task control	.006^c^
16.35 (7.10, −9.25)	(−34, 3, 4)	Insular cortex, L (4%)	CO task control	.007^b^
19.42 (8.28, −11.15)	(47, −50, 29)	Angular gyrus, R (56%)	Default mode	.009

*Note:* Absolute agreement difference (average of absolute value per node) and its decomposition into positive and negative contributory factors are listed on the first column. Nodes are labeled with the probabilistic Harvard‐Oxford cortical and subcortical structural atlas, using peak coordinate for each ROI.

Abbreviations: CO, cingulo‐opercular; DMN, default mode network; FP, frontoparietal; HOA, hip OA; KOA, knee OA; MNI, Montreal Neurological Institute; OA, osteoarthritis.

^a^ Network assignment in accordance with Figure 5.

^b^ *p*‐Values are one‐sided and calculated after randomly permutating participants over 1,000 iterations and generating a null model for reorganization estimates (agreement difference matrix).

^c^ Nodal differences were further validated in the KOA holdout sample.

^d^ HOA group at *p* < .05 (Table S1).

## 4 DISCUSSION

Using a large sample of OA patients, together with a robust methodology, we sought to study brain functional network properties and their relation to the clinical properties of the disease. We demonstrate for the first time that (a) OA patients have a global reorganization of nodal centrality properties while preserving network global topology, as captured by the hub disruption indices; (b) at a finer scale, we could identify a shift in the hierarchy of hub nodes. Primary sensory, motor, and parahippocampal regions become hubs; there is a large difference in connectivity between healthy subjects and OA patients, where these regions show high connectivity in KOA. On the other hand, the insula, a multimodal association area loses its centrality properties in KOA. (c) At a subnetwork, or community level, we showed that while brain networks can be equally well decomposed, the modular identity of multiple regions is rearranged in OA, in particular middle frontal gyrus, insula, and cingulate cortex. Finally, (d) we found that the defining feature of OA, pain intensity, is related to the interrelationship of the nodal degree of multiple brain regions in KOA and this result generalized to HOA.

### 4.1 Global topological properties

Global topological network properties were conserved in OA patients, as observed in earlier studies of chronic pain (De Pauw et al., 2020; Mansour et al., 2016). As Achard et al. (2012) expound, given that human brain networks have qualitatively similar global properties to those of other small world networks (Eguíluz, Chialvo, Cecchi, Baliki, & Apkarian, 2005), the preservation of these properties even in disease is expected. As long as the general architecture of the network is preserved, its global topological properties can be conserved, even when the system undergoes large changes.

### 4.2 Hub disruption indices

Consistent with the latter concept, we found local disruption in the order of importance of cortical nodes, calculated globally as hub disruption indices (*K*), both at the group and individual level. The local nodal connectivity properties counterbalances the increases and decreases throughout the brain resulting in an overall shift (*K*), while the overall mean property remains invariant (Achard et al., 2012). Interestingly, the magnitude of this disruption, about 20% when compared to the normative state, is similar to that seen in other chronic pain conditions (Mansour et al., 2016). In comatose patients, this shift is much larger (80%) (Achard et al., 2012) reflecting a more dramatic rebalancing of local connectivity.

### 4.3 Localized hub topology alterations

Although *K* indices were statistically significant in our discovery group, when testing across multiple link densities in our hold out sample (KOA and HOA), only centrality properties validated. Previous data showed regions which express aspects of node centrality (e.g., degree, larger number of edges), defined as hubs, tend to be more vulnerable to local perturbations (Stam, 2014). Remarkably, when we studied the nodal disruption profile at a finer‐grained level, we observed that the reduction in functional connectivity happened at the insula, a high‐order associative area. On the other hand, there was an increased centrality at the somatosensory cortex (M1/S1) and right fusiform gyrus/parahippocampal gyrus, the latter an area previously identified to predict drug analgesia in OA (Tétreault et al., 2016). This phenomenon, a selective loss of connectivity in highly central hub nodes accompanied with gain in degree in peripheral nodes seems to be a consistent finding in network studies of multiple neurological disorders (Crossley et al., 2014; Stam, 2014). An explanatory theory is provided by Stam (2014): in an initial phase of disease there is a rerouting of traffic from failing nodes to higher hierarchical nodes, which in time become overloaded. Eventually, in the chronic phase, peripheral (minimally connected) nodes become hubs, thus compensating for the overload of the failing hubs. It is not clear the extent to which our current results support this concept. All nodes that shifted their hubness are brain regions most likely involved in the OA pain state. It is possible that observed response is maladaptive, according to the *Stam* theory, where insula connectivity changes secondarily control rerouting of information and outgrowth of new connections to S1/M1. Alternatively, the decreased connectivity of the insula together with increased connectivity of S1/M1 and parahippocampus can reflect a coping process, diminishing the influence of the pain in the cortex, and increasing attention to SM control. At this point, we do not know the specific mechanisms or direction of influence regarding the observed shifts hub properties.

Although we were able to reliably isolate specific patterns underlying network disruption, there was an important finding that deserves further discussion: as also seen before (Mansour et al., 2016), by recalculating the *K* indices using only 20% of the nodes (randomly over multiple trials), we showed that nodal rank order disruption reflects altered connectivity that permeates the whole brain and is not merely driven by changes to specific regions. Most likely this is a reflection of the extent of stress that living with OA imposes on information processing throughout the brain (an overall decrease in high connectivity compensated by increase in low connectivity regions, diminishing the ability of information sharing, and thus the efficiency of cognitive processing).

### 4.4 Modular reorganization

Modular reorganization of brain networks allows us to evaluate network architecture at an intermediate level of organization, between global and local properties. In the past our group evaluated module allegiance (probability of a given node to be located in the same functional community of HC) in different chronic pain conditions, identifying the insular cortex and lateral parietal cortex (part of the SM network and DMN) as showing the largest variability in community membership. Mano et al., applying the same analytical approach in chronic back pain patients as we do here, identified mainly nodes located in the SM cortex (Mano et al., 2018). In our dataset, changes in modular identity were seen in the middle frontal gyrus, insula, and cingulate cortex, part of frontoparietal and cingulo‐opercular networks (validated in KOA and/or HOA) (Power et al., 2011). Why these regions are inconsistent between studies is not obvious. The rerouting of nodal information should affect the hierarchical modular organization of a network; thus, it is not surprising that the insular cortex shows modular reorganization. Middle frontal gyrus, part of the frontoparietal network, is highly related to task‐control, serving to initiate new task states by flexibly interacting with other control and processing networks (Marek & Dosenbach, 2018). Still, the across study variability of this outcome raises issues as to behavioral and physiological parameters that control modular reorganization, and future studies are needed to better clarify underling mechanisms.

### 4.5 Relationship between graph indices and OA clinical properties

In our study there was no strong association between *K* indices and clinical variables, in contrast to previous reports, where the hub disruption indices in chronic pain were related to pain intensity (Crossley et al., 2014; Mansour et al., 2016) or to other clinical key properties of disease (De Pauw et al., 2020). In another study, we also showed that for low back pain associated with disk herniation, *K*_D_ association with pain intensity was limited to males with high education (Huang et al., 2019). Thus, there are across study variations in the extent to which *K* indices relate to clinical parameters. Given that OA is a heterogeneous pathology with multiple clinical phenotypes (Deveza et al., 2019), it is likely that subgrouping patients with more in‐depth phenotyping could shed light into the relationship between *K* indices and clinical parameters.

When examining brain nodal degree properties, we could identify a set of nodes and their interrelationships reflecting OA pain intensity. This result was validated in our KOA holdout sample and generalized to the HOA sample. Regions in this model were located both in cortical and subcortical areas, across multiple functional networks. These results are consistent with our recent observation regarding brain structural properties and their relationship with clinical pain in OA, where we observed distributed regional changes, related in the latter case to the neuropathic pain in OA (Barroso et al., 2020). The causality of this relationship remains unclear, yet we showed that the distributed set of nodes and their information sharing with the rest of the brain relates to OA pain. If the OA pain is presumed to be dominantly reflecting nociceptive signals from the injured joint, then one would expect to observe a more circumscribed set of nodes reflecting related pain perception, and perhaps primarily the insula and S1 regions, according to prevailing concepts in the field (Malfait & Schnitzer, 2013). Our results instead suggest that distributed circuits throughout the brain contribute to OA pain and corroborate previous research showing OA pain may be better associated with brain regions involved in emotional and cognitive processing (Kulkarni et al., 2007; Parks et al., 2011).

### 4.6 Limitations

Some important limitations of the present study should be acknowledged. First, we use a single parcellation scheme to study nodal functional connectivity (Power et al., 2011). The hub disruption indices are known to be resilient to different parcellation schemes (Achard et al., 2012; Mansour et al., 2016); however, nodal graph properties are sensitive to distinct parcellations schemes, which could account for differences across studies (Fornito, Zalesky, & Bullmore, 2010). Another important technical limitation is the network link thresholding, a necessary step to use binary graphs. There is no current consensus on what threshold to use. Although global properties and graph disruption indices were stable across several correlation thresholds, caution should be used when interpreting results from binary graph models (Garrison, Scheinost, Finn, Shen, & Constable, 2015). In the modularity analysis, the choice of parameters for coupling strength and resolution {ω, γ} is nontrivial, and there is no current consensus (Sporns & Betzel, 2016). Finally, it is important to restate that since the control group was not large enough to be divided, it was used both in the discovery and validation analysis, therefore introducing some uncontrolled bias. Finally, the HOA group was small relative to our KOA group; thus, all HOA analyses were limited for validating/generalizing results obtained in KOA.

## 5 CONCLUSIONS

We demonstrated that OA pain is associated both with the disruption of whole‐brain and local functional connectivity. Although major nodal connectivity changes were identified in the S1/M1 regions, parahippocampal gyrus and the insula, the functional reorganization seemed to have propagated and eventually percolated throughout the whole brain, possibly reflecting the cost of the disease on information sharing across the brain. Pain intensity, a primary clinical concern in OA, was localized to distributed nodal functional connectivity changes in KOA, and this result strongly generalized for pain intensity in HOA. Therefore, our results dissociate the major hub disruptions from network connectivity related to OA pain, challenging the extent of dependence of OA pain on nociceptive signaling.

## CONFLICT OF INTEREST

The authors declare no conflict of interest.

## ETHICS STATEMENT

All procedures performed in studies involving human participants were in accordance with the ethical standards of the institutional research committee and with the 1964 Helsinki Declaration and its later amendments. Informed consent was obtained from all individual participants involved in the study.

## Supporting information


**Appendix** **S1:** Supplementary Information

## Data Availability

The data that support the findings of this study are available from the corresponding author upon reasonable request.
