# Supplementary material for: Reorganization of functional brain network architecture in chronic osteoarthritis pain
Source: Hum Brain Mapp. 2020 Nov 19;42(4):1206–22. doi: 10.1002/hbm.25287 (PMC7856636; doi:10.1002/hbm.25287)
Supplement: Supplementary file 1 — Appendix S1: Supplementary Information [file HBM-42-1206-s001.docx]

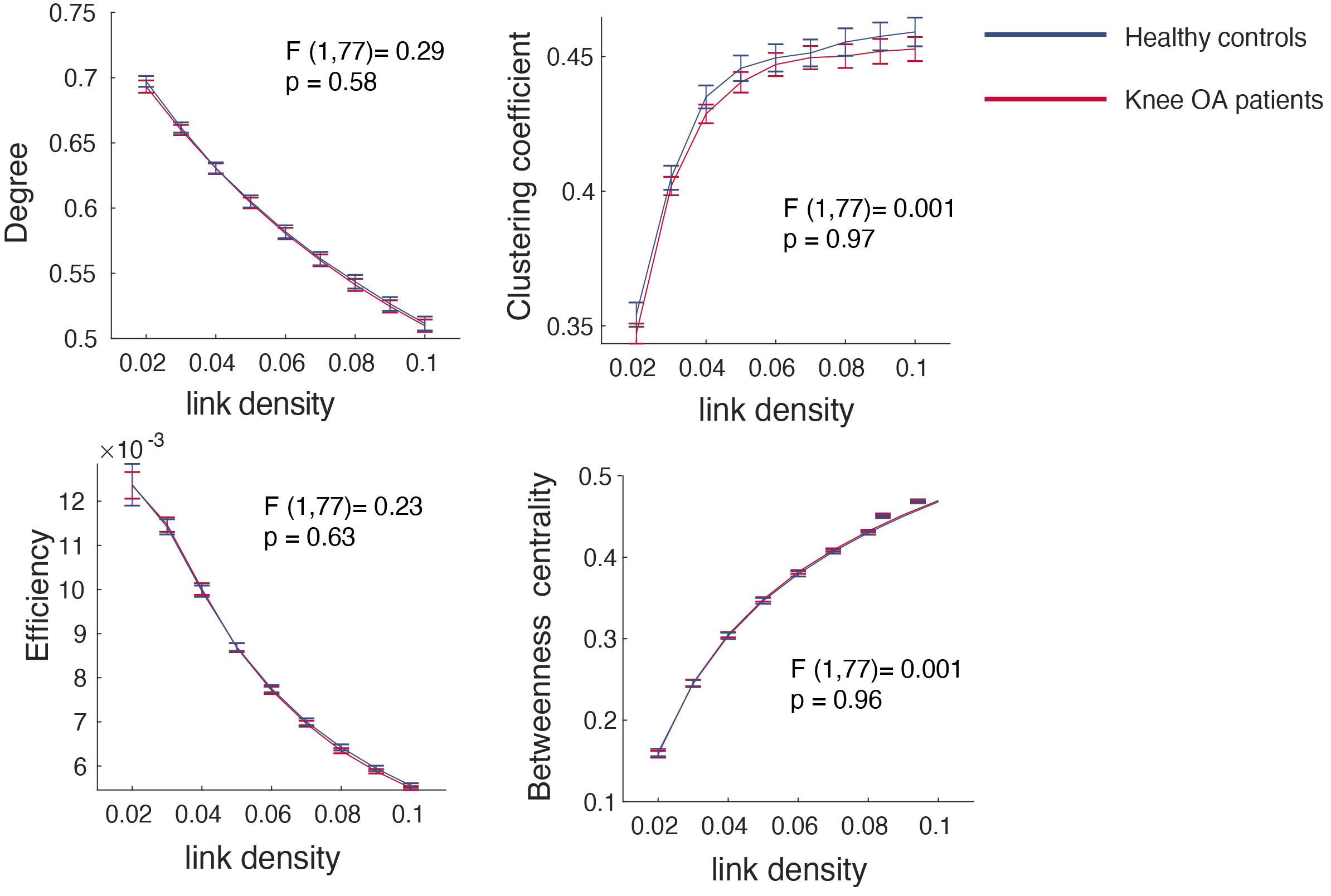


**Figure S1.** **Global graph topological properties of brain networks do not differentiate between OA patients (red) and healthy controls (blue).** Global network topology measures (global efficiency, clustering coefficient, betweenness centrality and modularity, small-world-ness) do not show statistically significant differences between groups (repeated measures ANCOVA, controlling for effects of age and sex; data plotted as mean ± s.e.m). HC, healthy control; KOA, knee osteoarthritis.


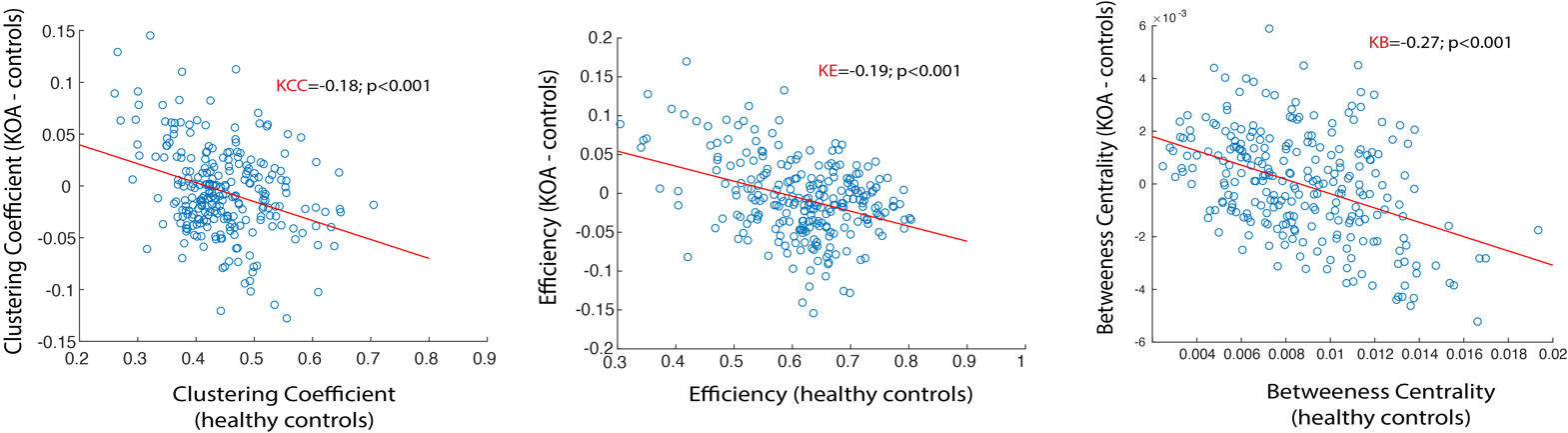


Figure S2. Group averaged hub disruption indices for nodal properties: Clustering coefficient (K_CC_), Efficiency (K_E_) and Betweenness Centrality (K_B_). For all properties, K indices, calculated at 5% link density, correspond to the slope of the red line (regression coefficient from a linear model fitting the cloud of points). All indices show a significant negative coefficient (KCC=-0.18; KE=-0.19; KB=-0.27), representing for each nodal property, a rank order disruption: loss for highest ranked regions in the control group, and concurrently a gain for the lower rank regions in the OA group.


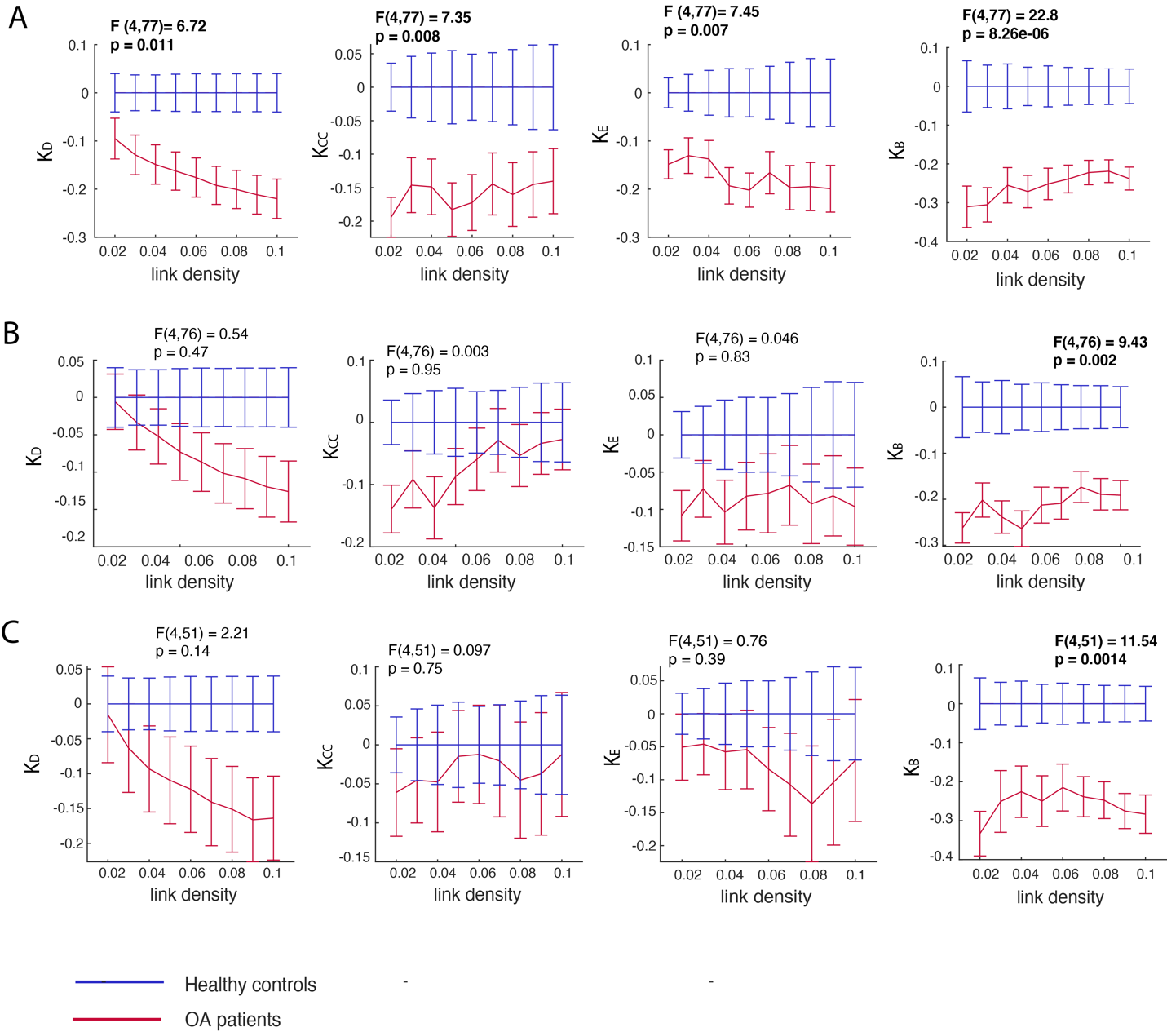


**Figure S3.** **Hub disruption indices in KOA are robust to network connection density; results validate across all link densities for K_B_** **in KOA and HOA hold-out groups**. **A.** Results for repeated measures ANCOVA controlling for age and gender at 9 different link densities (0.01:0.01:0.1%) in the KOA discovery group show *K* indices are robust to network link threshold. **B and C.** show the same analysis in the hold-out, KOA (B.) and HOA (C.) testing groups: only K_B_ index validates across all link densities; K_D_ shows a similar pattern, with larger differences arising at higher network link densities. K_D_: hub disruption index of degree; K_BC_: hub disruption index of betweenness centrality; K_E_: hub disruption index of efficiency; K_CC_: hub disruption index of clustering coefficient. Data is plotted as mean ± s.e.m


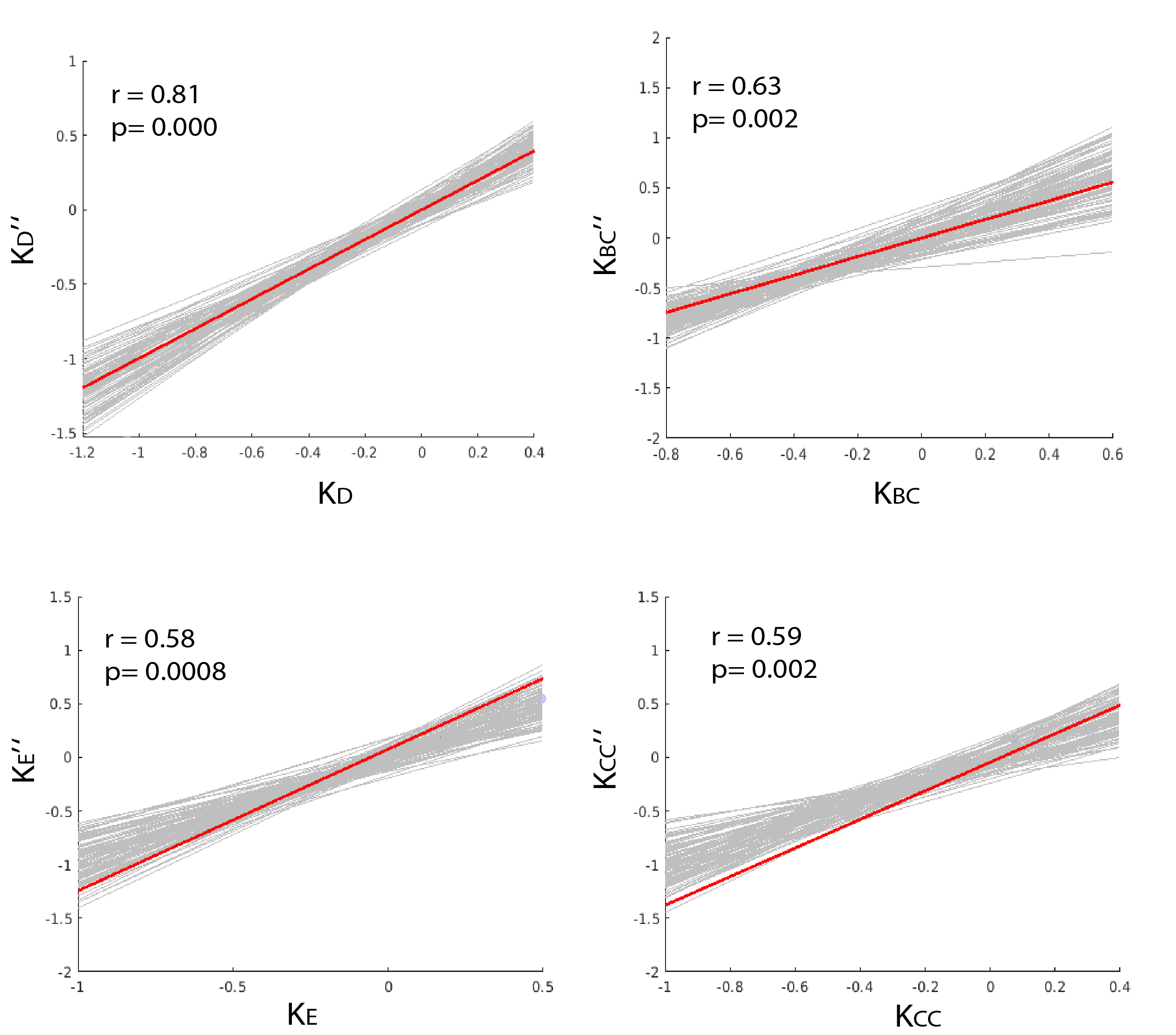


**Figure S4.** **Hub disruption indices reflect global altered connectivity**. K’’ is calculated after a random removal of 80% nodes, i.e. from a random 20% subset of nodes Degree, Clustering Coefficient, Efficiency and Betweenness centrality for the KOA patients, and this process repeated over 100 times. The correlation between K and K'' varied each time to some extent, presumably due to a small number of the total number of ROIs (n=56). The shades and the slopes in gray depict an empirical 95% confident interval derived from the iterations, and the in red depict the average correlation. * p<0.05.


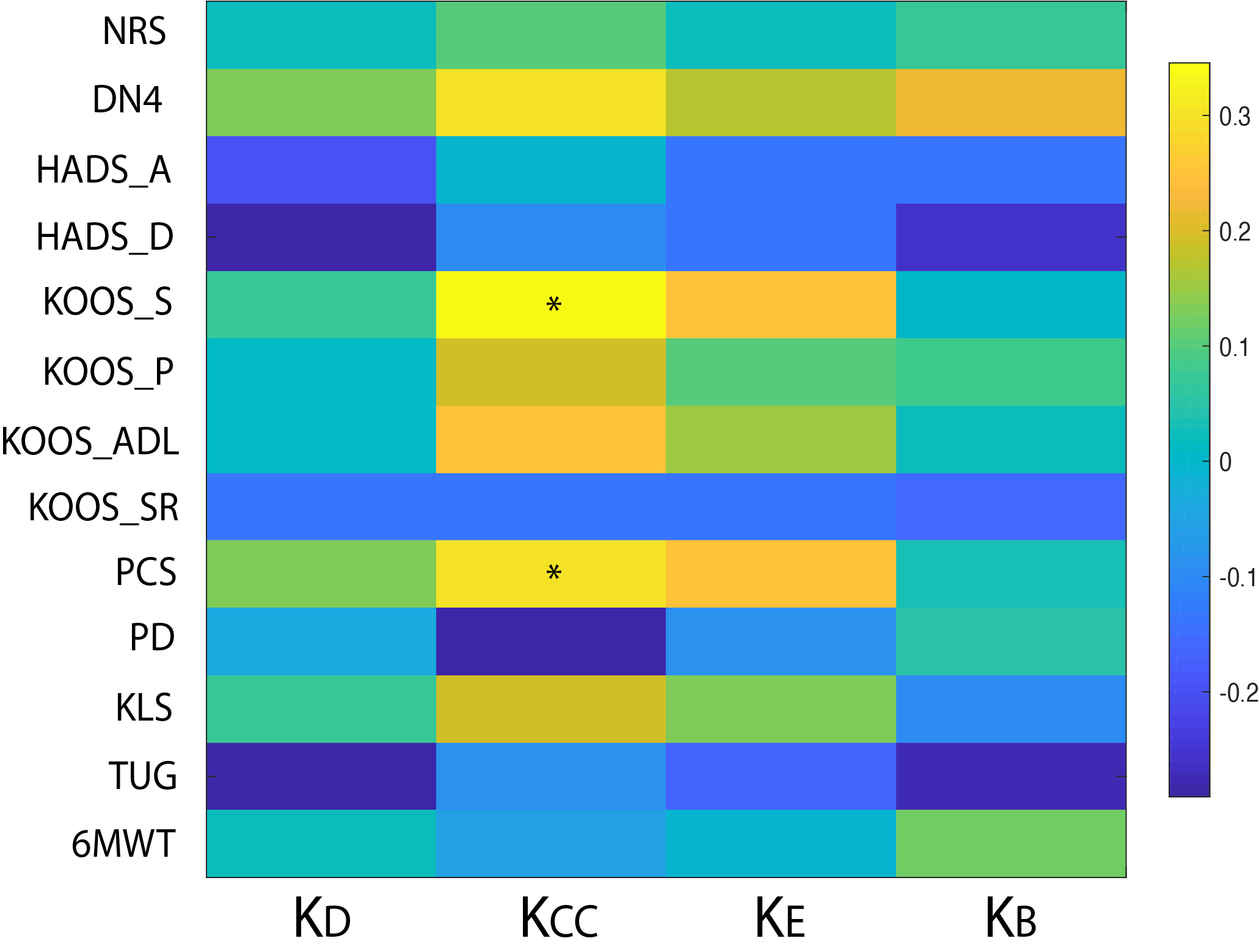


**Figure S5.** **Lack of association between hub disruption indices and clinical variables.** Partial correlations while controlling for age and gender were performed with 13 clinical variables of importance in KOA. All values were |r|<0.33. Only two correlations reached statistical significance at p<0.05: KOOS_S, KOOS symptoms subscale (r=0.33; p=0.021) and PCS, pain catastrophizing scale (r=0.3; p=0.045), with K_cc_. These correlations did not survive multiple comparisons. K_D_: hub disruption index of degree; K_BC_: hub disruption index of betweenness centrality; K_E_: hub disruption index of efficiency; K_CC_: hub disruption index of clustering coefficient.

| Coordinate in MNI Space | Harvard-Oxford Cortical and Subcortical Structural Atlas | Network Assignment ^(1)^ | p value * validation  (KOA/HOA) |
| --- | --- | --- | --- |
| (44, -53, 47) | ﻿Angular Gyrus, R (40%) | FP Task Control | 0.06^$^ // 0.14 |
| **(32, 14, 56)** | **﻿Middle Frontal Gyrus, R (42%)** | **FP Task Control** | **0.02* // 0.05*** |
| (55, -44, 37) | ﻿Supramarginal Gyrus, R (43%) | Salience | 0.32 // 0.52 |
| (48, 25, 27) | ﻿Middle Frontal Gyrus, R (39%) | FP Task Control | 0.15 // 0.3 |
| **(39, 18, 39)** | **﻿Middle Frontal Gyrus, R (50%)** | **FP Task Control** | **0.04* // 0.08^$^** |
| (-60, -25, 14) | ﻿Parietal Operculum Cortex, L (35%) | Auditory | 0.58 // 0.9 |
| **(-5, -18, 34)** | **﻿Cingulate Gyrus, L (29%)** | **CO Task Control** | **0.61 // 0.02*** |
| **(-34, 3, 4)** | **Insular Cortex, L (4%)** | **CO Task Control** | **0.01** // 0.2** |
| (47, -50, 29) | ﻿Angular Gyrus, R (56%) | Default Mode | 0.1 // 0.39 |

**Table S1. Validation of brain regions showing modular reorganization after permutational based analysis against a random model, at a cut-off threshold of p<0.01.** Absolute agreement difference (average of absolute value per node) and its decomposition into positive and negative contributory factors is listed on the first column. Nodes are labeled with the probabilistic Harvard-Oxford Cortical and subcortical structural atlas, using peak coordinate for each ROI. *p values are one-sided and calculated after randomly permutating participants over 1000 iterations and generating a null model for reorganization estimates (agreement difference matrix). MNI, Montreal Neurological Institute; CO, Cingulo-opercular; FP, Frontoparietal.
